# Investigation of Corrosion Behavior of Oxygen-Free Copper Canisters in Groundwater Chemistry of Deep Geological Repositories

**DOI:** 10.3390/ma17010074

**Published:** 2023-12-22

**Authors:** Tae Ho Yun, Taeyong Kim, Seunghyun Kim, Jisoo Kim

**Affiliations:** 1 Department of Precision Mechanical Engineering, Kyungpook National University (KNU), 2559 Gyeongsang-daero, Sangju-si 37224, Gyeongsangbuk-do, Republic of Korea; thyun87@knu.ac.kr; 2 Department of Advanced Science and Technology Convergence, Kyungpook National University (KNU), 2559 Gyeongsang-daero, Sangju-si 37224, Gyeongsangbuk-do, Republic of Korea; taey.kim.0221@gmail.com; 3 Joining Technology Department, Korea Institute of Materials Science (KIMS), 797 Changwon-daero, Changwon-si 51508, Gyeongsangnam-do, Republic of Korea

**Keywords:** canister, high-temperature deep geological disposal, Korean groundwater, electrochemical impedance spectroscopy (EIS)

## Abstract

The disposal of nuclear waste represents a paramount concern for human safety, and the corrosion resistance of containers within the disposal environment stands as a critical factor in ensuring the integrity of such waste containment systems. In this report, the corrosion behavior of copper canisters was monitored in Olkiluoto-simulated/-procured groundwater (South Korea) with different temperatures. The exposure of copper in the procured groundwater at 70 °C revealed a 3.7-fold increase in corrosion vulnerability compared with room temperature conditions, with a current density of 12.7 μA/cm^2^. During a three-week immersion test in a controlled 70 °C chamber, the canister in the Korean groundwater maintained a constant weight. In contrast, its counterpart in the simulated groundwater revealed continuous weight loss, indicating heightened corrosion. An X-ray diffraction (XRD) analysis identified corrosion byproducts, specifically Cu_2_Cl_3_(OH) and calcite (CaCO_3_), in the simulated groundwater, confirming its corrosive nature. The initial impedance analysis revealed distinct differences: Korean groundwater exhibits high pore resistance and diffusion effects, while the simulated groundwater shows low pore resistance. Consequently, the corrosion of copper canisters in the Korean environment is deemed relatively stable because of significant differences in ion concentrations.

## 1. Introduction

Nuclear energy, prized globally for its cleanliness and efficiency, has spurred vigorous development efforts worldwide. However, with this progress comes the pressing challenge of managing the substantial volume of radioactive nuclear waste it generates. The internationally accepted solution for treating high-level nuclear radioactive waste (HLRW) is deep geological disposal, involving its burial at depths ranging from 500 to 1000 m underground for long-term containment [1]. This disposal method follows a multi-barrier approach, wherein the HLRW resides within a container. This container is typically secured as an engineering barrier system (EBS), comprising an overpack (canister), buffer, backfill, and repository walls [2]. Sometimes, these components are further enveloped by the natural geological barrier provided by the surrounding rock cluster.

As part of ensuring the safety of the EBS, HLRW repository safety assessments are fundamentally required. The objective of these assessments is to secure the system’s safety when each component of the engineered barrier is exposed to an environment similar to the actual disposal environment. Consequently, various countries have conducted long-term corrosion assessments for candidate materials in deep geological disposal environments, such as anoxic conditions. However, such research has not yet been conducted in South Korea.

In an EBS, carefully selecting the outer layer material is essential to ensure safety. Beyond possessing chemical resistance, the canister must exhibit robust mechanical strength to endure the pressures induced by hydrostatic pressure and the swelling of bentonite clay buffer. Candidates for the canister should adhere to heat and radiation exposure limits in the near field while ensuring that the selected materials do not undermine the effectiveness of the adjacent buffer. To adhere to these criteria, the maximum surface temperature of the canister has been allowed to be 100 °C [2]. The outer-layer material typically employs metallic canisters [3]. Consequently, comprehending and accurately predicting the corrosion behavior of the materials used in these canisters assumes paramount importance, especially considering the requirement for these components to maintain their structural integrity for a minimum of 100,000 years [4].

Several candidate canister materials have been proposed, such as nickel alloys, stainless and carbon steels, titanium alloys, and copper [2,5,6,7,8]. Some canister candidates, such as austenitic stainless steel, Ni-Cr-Mo alloys, and Ti alloy, possess a corrosion-inhibiting mechanism by forming a passive film in groundwater environments. Despite their remarkable resistance to general corrosion, these alloys are susceptible to localized corrosion risks, notably pitting (e.g., in Cl^−^ environments). On the other hand, carbon steel and oxygen-free copper are prone to high corrosion vulnerability in oxidizing environments. Among them, oxygen-free copper (OFC) is one of the promising candidates owing to its outstanding benefits, such as cost-effectiveness, minimizing segregation at grain boundaries, and increasing creep resistance in the case of phosphorous-doped ones [9]. Moreover, OFC demonstrates thermodynamic stability in anaerobic, reducing environments [3,5]. OFC has been applied in the KBS-3 multi-barrier disposal system in Sweden and Finland. South Korea has a growing interest in oxygen-free copper as a canister in EBSs for deep geological disposal. Copper has been frequently recommended for utilization as a HLRW container, especially when combined with densely compacted bentonite backfill in repositories with chloride-dominated groundwaters [10]. Although copper has traditionally been linked to host rocks with higher permeability for extended container lifetimes, it is equally applicable in less permeable sedimentary formations. Its suitability extends to saline environments, where chloride ions facilitate copper’s active dissolution, mitigating localized corrosion and Stress Corrosion Cracking (SCC) [10]. The following equation represents the general corrosion of copper in oxygen-chloride-containing compacted bentonite [11]:

(1) $$Cu↔CuCl_{ADS}↔CuCl_{2}^{-}$$

(2) $$CuCl_{2}^{-}\to {Cu}^{2+}\to CuCl_{2}\cdot 3Cu{\left(OH\right)}_{2}(bentonite-Cu\;interface)$$

(3) $$CuCl_{2}^{-}\to {Cu}^{2+}\underset{J_{Cu\left(II\right)}}{\to }Cu^{2+}(bentonite-Cu\;interface)$$

(4) $$Cu^{2+}\to CuCl_{2}^{-}\;|\;Fe\left(II\right)\to Fe\left(III\right)\left(bentonite-Cu\;interface\right)$$

(5) $$CuCl_{2}^{-}↔Cu_{2}O$$

In oxygen-containing systems, the cathodic reduction of O_2_ plays a pivotal role in supporting copper dissolution, forming cuprous-chloride complexes (and CuCl_3_^2−^ at elevated chloride concentrations). These species are contingent on diffusion to and from the material surface. These corrosion products do not provide highly protective, permitting continued corrosion of the underlying copper surface. Plus, as corrosion progresses, O_2_ is gradually consumed. Initially, corrosion occurs under aerobic conditions, but in later stages, anaerobic conditions prevail, leading to significantly reduced corrosion rates [10]. Sulfide ions also play a significant role as ions that influence copper corrosion. The prevailing assumption is that the corrosion of the container will be regulated by the movement of this sulfide, either originating from or passing through the clay buffer, to reach the surface of the copper container [1]. The reaction between copper and sulfide ions is known to involve two main reactions [10]:

(6) $$Cu+{HS}^{-}\to Cu{\left(HS\right)}_{ADS}+e^{-}$$

(7) $$Cu+Cu{\left(HS\right)}_{ADS}+{HS}^{-}\to {Cu}_{2}S+H_{2}S+e^{-}$$

Reaction (6) represents a fast one-electron transfer process, while Reaction (7) represents a second slow one-electron process. In an anaerobic solution containing Na_2_S and NaCl, the corrosion product formed on a copper surface took the form of a single-layered compact Cu_2_S film [12]. Once this compact film was established, the growth process was primarily governed by the diffusion of Cu^+^ within the film. However, previous research has indicated that the characteristics of the sulfide film formed on copper are influenced by the solution concentrations of both sulfide and chloride [13]. In solutions with a high chloride-to-sulfide concentration ratio, the sulfide film exhibited a cellular structure.

As previously discussed in the previous paragraphs, the corrosion behavior of canisters is intricately linked to the specific groundwater environmental conditions within the repository [14]. It is noteworthy that groundwater composition exhibits substantial variations across diverse geological formations. Therefore, in many studies, experiments are conducted using synthetic groundwater or solutions containing chloride and sulfide instead of actual groundwater. A study has explored the suitability of different materials in room-temperature Korean groundwater. However, from our minimal perspective, a comprehensive analysis of corrosion behavior should consider the elevated temperatures (ranging from 100 to 200 °C) likely encountered during the actual disposal process. Examining corrosion under these heightened temperature conditions is imperative for a more accurate understanding of canister materials’ long-term performance and integrity in nuclear waste repositories. In particular, most of the corrosion studies conducted on copper in groundwater environments have been based on deep geological repositories in countries such as Sweden and Finland. Therefore, evaluating corrosion in the context of South Korea’s groundwater environment is a critical factor for future safety assessments of deep geological repositories.

To assess the corrosion characteristics of OFC canister materials within the naturally aerated groundwater of an underground research facility with a depth of 210 m, this study employed direct potentiodynamic polarization (PDP) and indirect electrochemical impedance spectroscopy (EIS) analyses to examine the polarization and kinetic corrosion behaviors. Additionally, scanning electron microscopy (SEM), energy dispersive spectrometry (EDS), and X-ray diffraction pattern (XRD) techniques were utilized to investigate the corrosion products. The uniformity of the corrosion layer was explored through cross-sectional observations via a white interferometer measurement. Comparative corrosion rates were determined through high-temperature immersion experiments and weight gain/loss measurements.

It is crucial to note that disposal processes in groundwater environments are inherently anaerobic, have distinct corrosion mechanisms, and are time-consuming processes. The final objective of our research group was to evaluate the corrosion behavior of copper canisters within such anaerobic settings. As a preliminary step, we sought to conduct a relative assessment under aerated conditions to simulate a high-temperature disposal scenario.

## 2. Materials and Methods

In the preparation of artificial groundwater (AG) in the Olkiluoto region, a range of chemical substances was employed. Specifically, calcium chloride hexahydrate (98.0%), ammonium chloride (99.99%), sodium hydrosulfide hydrate, potassium chloride (99%), and sodium sulfate (99%) were sourced from Sigma Aldrich (St. Louis, MO, USA). Magnesium chloride hexahydrate (98%) was procured from Samchun Pure Chemicals Co., Ltd. (Seoul, Republic of Korea), while sodium chloride (pure salt grade) was obtained from Ascott Analytical (Tamworth, UK). Otherwise, no further purification or post-treatment measures were undertaken for procured groundwater (PG) utilization in South Korea.

The OFC test specimen was sheet-type (2 × 2 × 3 cm^3^). Prior to all the experiments, the specimen was ground with silicon carbide sandpapers (from 1000 to 2000 grits), surface-finished with 0.6 μm diamond paste, and cleaned several times with deionized water, ethanol, and acetone.

The procedure for crafting artificial groundwater in the Olkiluoto unfolded in the following sequence [15]: Initially, a 5 L beaker accommodated the introduction of CaCl_2_, NH_4_Cl, Na_2_SO_4_, MgCl_2_, and NaCl, alongside 4 L of deionized water. Vigorous stirring was conducted for 1 h. Subsequently, the concocted solution underwent dilution using additional deionized water, culminating in producing a uniform artificial groundwater solution with a total volume of 25 L.

The as-prepared groundwater solution was placed within a fume hood to prevent additional oxygen intrusion and light-induced decomposition. The compositions of the referenced Olkiluoto groundwater, manufactured artificial groundwater, and collected groundwater are summarized in Table 1. In comparison to the Olkiluoto groundwater, the procured groundwater exhibited significantly lower concentrations of cations such as Na^+^ and Ca^2+^, at approximately 1/100 of the levels. Moreover, NH^4+^ was not detected, and the concentration of Cl^−^ was remarkably low, around 1/740 of the previous levels. Additionally, HS^−^ was not detected, and methane (CH_4_) was also absent in the procured groundwater. Considering these characteristics, it was anticipated that copper corrosion would minimally progress under South Korea’s groundwater conditions. For the electrochemical tests, the surface-finished copper specimen was equipped with the FSH2 flat sample holder (exposed area: ~1 cm^2^) to evade cut-edge corrosion. As-prepared AG and PG were used without purification in electrochemical tests like open circuit potential (OCP), PDP, and EIS.

For the weight gain/loss evaluation test, the surface-finished copper specimen was directly placed into a reservoir containing groundwater solution (AG or PG) at 70 °C, then sealed with a stopper. The reservoir was placed in a convection oven (Samheung Scientific Corp., Seoul, Republic of Korea) for three weeks. Also, the weight gain/loss measurement was conducted without any etching process. All OFC specimens employed in the various tests were denoted with labels, such as Cu-AG-L (representing copper specimens tested in artificial groundwater at a low temperature), Cu-AG-H (representing copper specimens tested in artificial groundwater at a high temperature), Cu-PG-L, and Cu-PG-H.

The electrochemical testing employed an OFC-equipped holder as the working electrode, with a three-electrode system utilizing a Hg/HgO reference electrode and a Pt-mesh counter electrode (17.5 cm^2^). The measurements, including OCP, PDP, and EIS, were carried out at either room temperature or 70 °C using a BioLogic VSP-128 potentiostat (Seyssinet-Pariset, France).

OCP measurements continued until the potential deviation remained under one mV/h, followed by EIS measurements performed at OCP with a ten mV sinusoidal perturbation within a frequency range of 10^−1^ to 10^6^ Hz, with 20 steps per decade. Subsequently, PDP measurements took place with a scan rate of 0.167 mV/s, spanning from −1 to 1.5 V_Hg/HgO_ versus the OCP.

The immersion experiments’ time-dependent changes in the samples were recorded using a Samsung Galaxy S8+ smartphone (Samsung Electronics, Suwon, Republic of Korea). To analyze the corrosion products, surface morphology, and elemental distribution, maps were acquired through SEM and EDS analyses (Regulus 8220, HITACHI, Tokyo, Japan). Moreover, XRD analysis (EMPYREAN, Malvern Panalytical, Malvern, UK) with Cu Kα radiation (λ = 1.5418 nm) was employed to identify corrosion products in detail. To analyze the corrosion product formation behavior during an immersion test, 50 wt% of aqueous nitric acid was used for the etchant. To assess the corrosion product formation on the OFC based on groundwater composition and temperature, surface topography was assessed through observations utilizing a white interferometer (NV-2700, Nano system Inc., Daejeon, Republic of Korea).

## 3. Results and Discussion

### 3.1. Corrosion Behavior of the OFP

**Figure 1:**
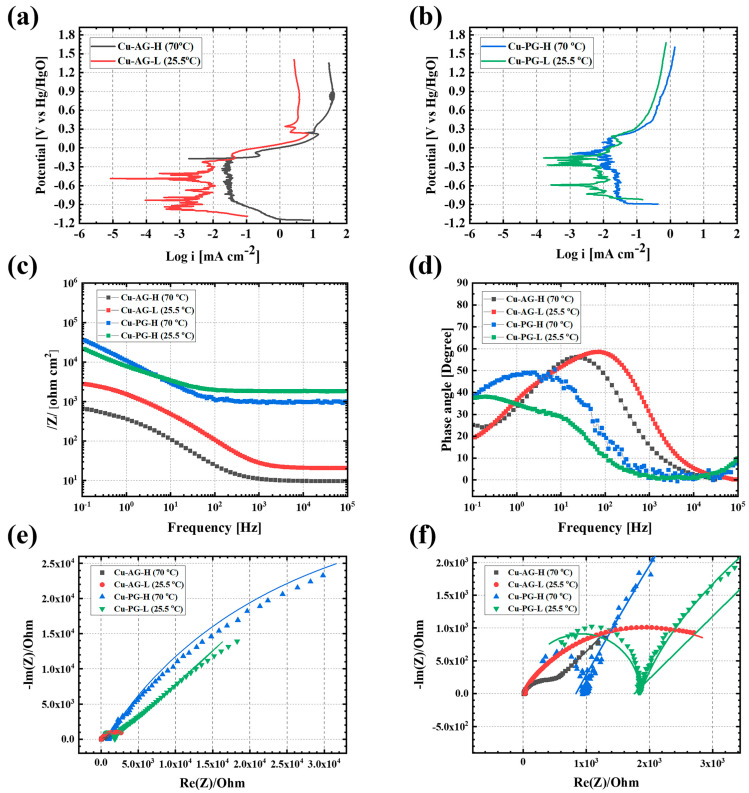
Electrochemical test result of oxygen-free copper (OFC) corrosion behavior in different temperature and groundwater types: (**a**) PDP curves in Olkiluoto-simulated artificial groundwater (AG), (**b**) PDP curves in procured groundwater (PG) in South Korea, (**c**) Bode plot of total impedance along with frequency, (**d**) Bode plot of phase angle along with frequency, (**e**) Nyquist plot, and (**f**) magnified version.

An electrochemical analysis was conducted to explore the corrosion behavior in relation to the groundwater type and temperature, with the results presented in Figure 1. Figure 1a,b reveal the PDP curve of the OFC following the stabilization of OCP. Notably, as the temperature of the AG corrosion environment rose, the corrosion potential exhibited an ascent from −0.49 to −0.17 V, concomitant with a concurrent rise in the corrosion current density, escalating from 2.9 to 30.90 μA/cm^2^. This signified a notably swift advancement in the corrosion process. Also, a tiny anodic/cathodic complex process was observed alongside the noise in the cathodic phase. This phenomenon, attributed to the cleaning of the working electrode and the reduction reaction of substances more active than copper in the solution, holds minor consideration as these substances eventually dissolve in the anodic reaction of copper (Figure 1).

Furthermore, the concentration polarization and limiting current density were evident in the cathodic process, typically occurring when the speed of the reduction reaction was exceptionally swift. This occurrence was influenced by diffusion layer thickness, the diffusion coefficient, and the solution concentration. Notably, at 70 °C, robust concentration polarization was observed due to the proportional relationship between the diffusion coefficient and temperature. Intriguingly, the anodic reaction process depicted in Figure 1a indicates re-passivation. However, considering the current density order, this was attributed to the temporary deposition of excessive corrosion products generated due to an accelerated corrosion process rather than any corrosion inhibition function.

In Figure 1b, the PDP results in the Korean-type groundwater at low temperatures demonstrated similar current density values to those in the artificial groundwater, indicating similar corrosion stability, as detailed in Table 2. At an elevated temperature of 70 °C, the current density in the PG was approximately 3.7 times greater, resulting in a correspondingly accelerated corrosion rate. However, the AG manifested a noteworthy surge of 10.7 times in current density, thereby indicating a markedly accelerated corrosion rate in comparison to the PG. This discernible contrast in corrosion rates appeared to be influenced by a significant distinction in the absolute quantity of ions within the solution, estimated to be within the range of 1/100 to 1/800. In the previously reported literature [5], the current density for OFC at low temperatures (room temperature) stood at 0.94 μA/cm^2^. The ion composition in the groundwater cited in the literature was approximately 1/2 to 1/7 of that found in our study’s groundwater. While the previous report exclusively relied on PDP results to present copper stability, our findings unveiled a significant contrast, with a current density of 3.56 μA/cm^2^ at low temperatures, indicating an approximately 3.8-fold difference. Furthermore, at elevated temperatures (70 °C), a substantial 14.1-fold difference in corrosion rates was evident. In light of the critical safety considerations regarding the disposal of high-level radioactive waste, the estimation of the canister’s lifespan may accordingly exhibit variations. Similar to the result in Figure 1a, Figure 1b represents an increase in current density with rising temperature, accompanied by the emergence of a limiting current region due to concentration polarization.

Figure 1c,d showcase Bode plots from the EIS measurements. Notably, the total impedance in the PG environment significantly exceeded that in the AG context, signifying heightened stability. Conversely, the AG environment displayed a twofold lower impedance and heightened susceptibility to corrosion with temperature factors. After analyzing the frequency domain of the phase angle change’s tipping point (Figure 1d), the AG case at high frequencies indicated influence from pore resistance (R_p_) and coating capacitance (C_c_). Conversely, the PG case demonstrated a turning point at lower frequencies, indicating the impact of charge transfer resistance (R_ct_), double-layer capacitance (C_dl_), and the Warburg component (W). Further insight from the Nyquist plot in Figure 1e revealed that the OFC exposed to the PG environment exhibited a substantial C_dl_|R_ct_-W value, indicating the application of a diffusion-controlled process. This phenomenon may have been associated with vacancy diffusion through the Cu_2_O film or the diffusion of Cu(II) to the surface. Cu(II) serves as an intermediate or secondary oxidant when copper undergoes corrosion, with O_2_ acting as the primary oxidant [10]. The equivalent circuit utilized for the EIS fitting and the OCP plot are presented in Figure S1. To ensure precise fitting, the capacitance was substituted with a constant phase element (CPE). Capacitances were determined through the formula [16]:

*C* = *Y_o_* (ω″_max_)^α−1^ (8)

where ω″_max_ denotes the frequency corresponding to the maximum of the −Z″ vs. ω dependence, a parameter independent of the CPE exponent α, C is the capacitance, and *Y_o_* is the frequency-independent admittance of the CPE.

**Figure 2:**
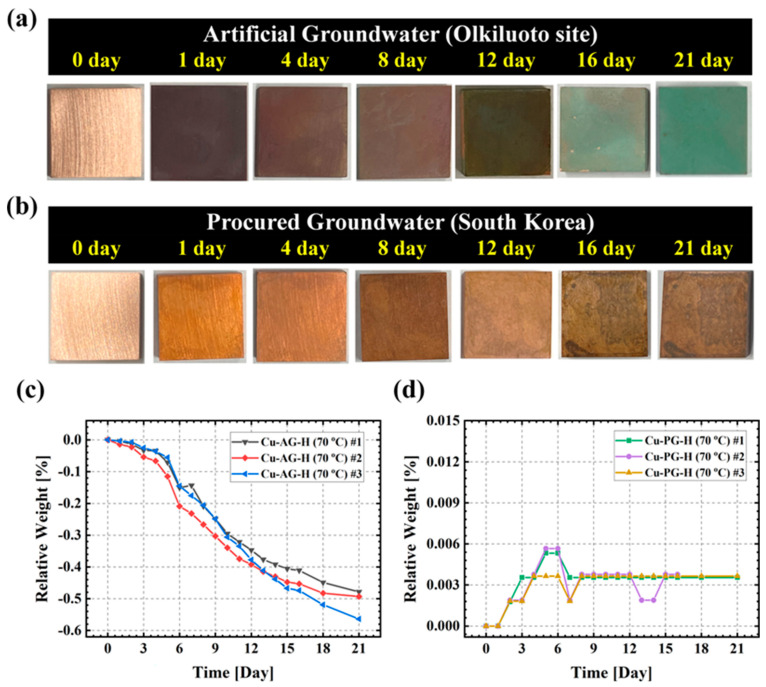
(**a**,**b**) Visual images of the OFC with an immersion test in AG or PG for three weeks; (**c**,**d**) weight gain/loss tendencies with the immersion test.

Subsequently, the outcomes of the immersion experiment conducted on the OFC sample at 70 °C are illustrated in Figure 2. The surface images of the specimens (Figure 2a,b) revealed that after 21 days, the surface exhibited a blue color (in the AG environment) and a darkish-brown hue (in the PG environment). Given that Cu ions manifest as blue, this observation indicated a notably swift corrosion rate for the OFC exposed to the AG environment. Additionally, within one day of immersion, the surface color transitioned to black, suggesting the formation of CuO. In contrast, the OFC exposed to the PG environment experienced a reduction in brightness after eight days and appeared dark brown, presumably indicating a mixture of Cu and CuO [17].

Figure 2c,d represent measurement data from weight gain and loss measurements. Three separate experiments were conducted to enhance precision and reproducibility, all yielding consistent results. Notably, no etchant was utilized during weight gain or loss measurements. In the AG environment, copper corrosion exhibited rapid decline, while in the PG environment, some weight gain was observed. In the AG environment, copper elution was significantly accelerated, while the PG environment witnessed some elution, which was moderated by stable corrosion products, resulting in a notably lower corrosion rate. Visual representations of the groundwater solution before and after the immersion test, with the immersion time indicated, can be observed in Figure S2.

Despite various reports that have been published about copper corrosion in different corrosive conditions, which are not in groundwater, our minimal perspective pointed to differences in overall ion content as a probable contributing factor, as expressed in Table 1. Further comprehensive investigation of these crucial factors is planned for subsequent follow-up research.

### 3.2. Optical Analysis of the OFC Corrosion Products

**Figure 3:**
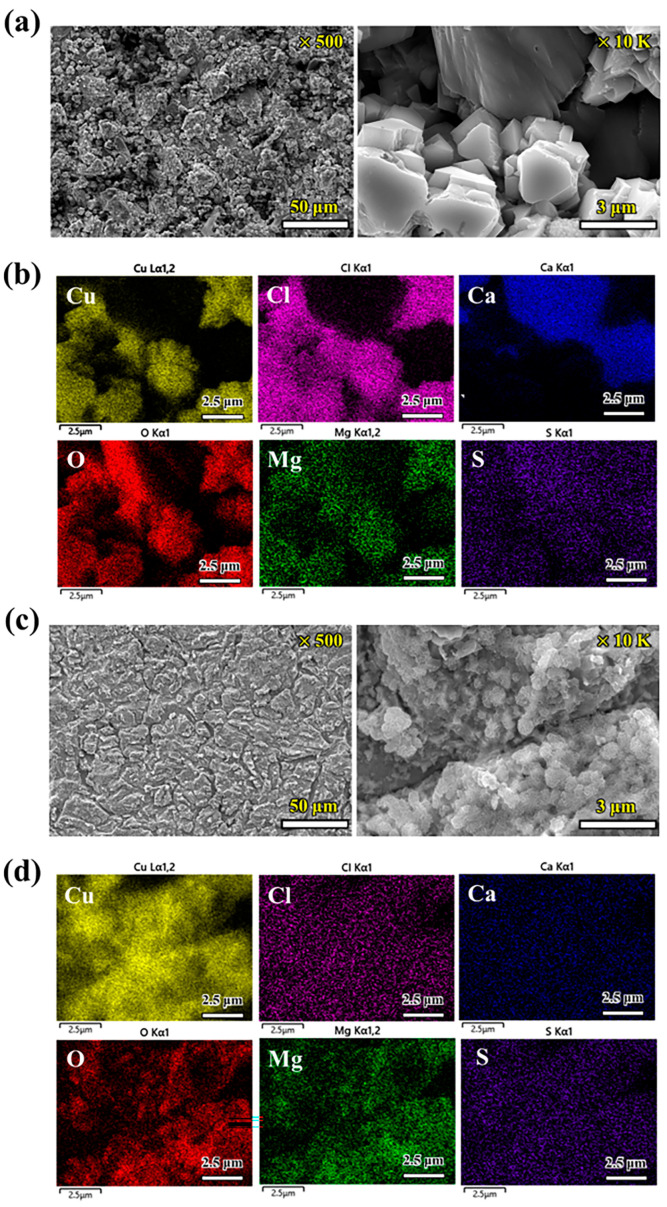
Scanning electron microscopy (SEM) and elements mapping with an energy dispersive spectrometry (EDS) analysis of the OFC exposed to AG at 70 °C: (**a**,**b**) surface layer without etching and (**c**,**d**) surface layer with etching; 50 wt% of aqueous nitric acid was used for the etchant.

**Figure 4:**
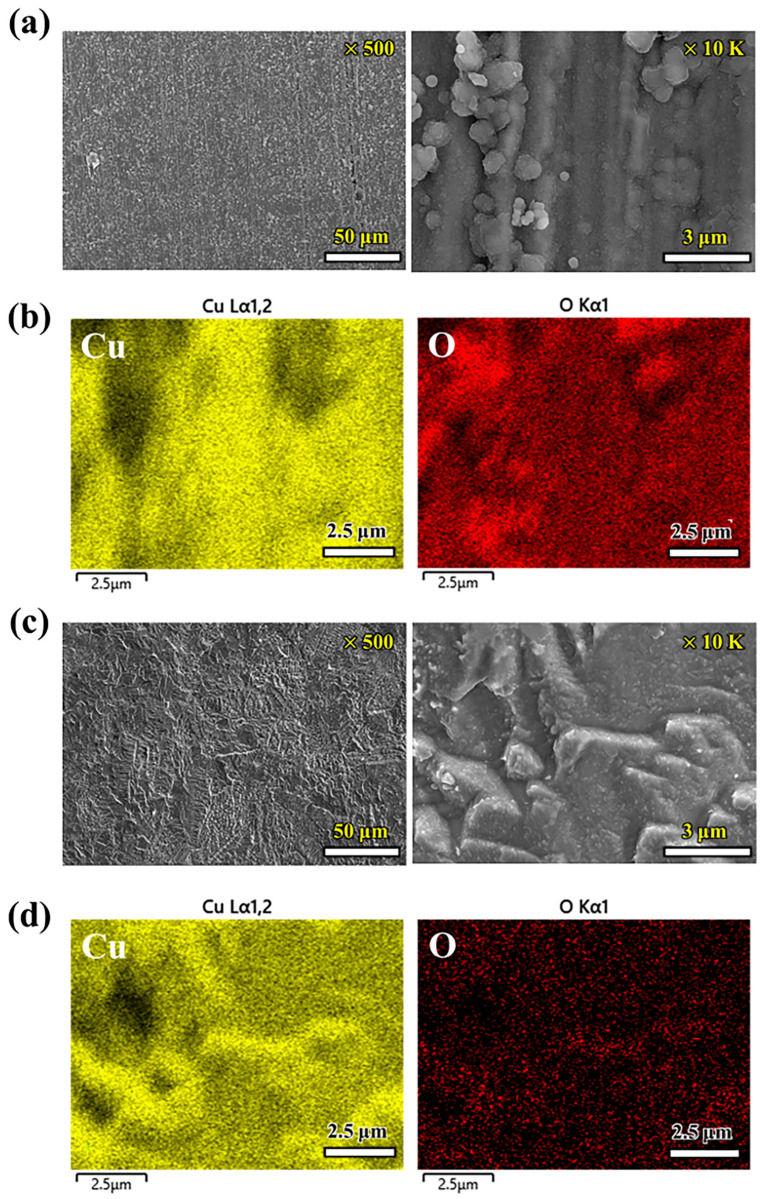
SEM and elements mapping with an EDS analysis of the OFC exposed to PG at 70 °C: (**a**,**b**) surface layer without etching and (**c**,**d**) surface layer with etching; 50 wt% of aqueous nitric acid was used for the etchant.

**Figure 5:**
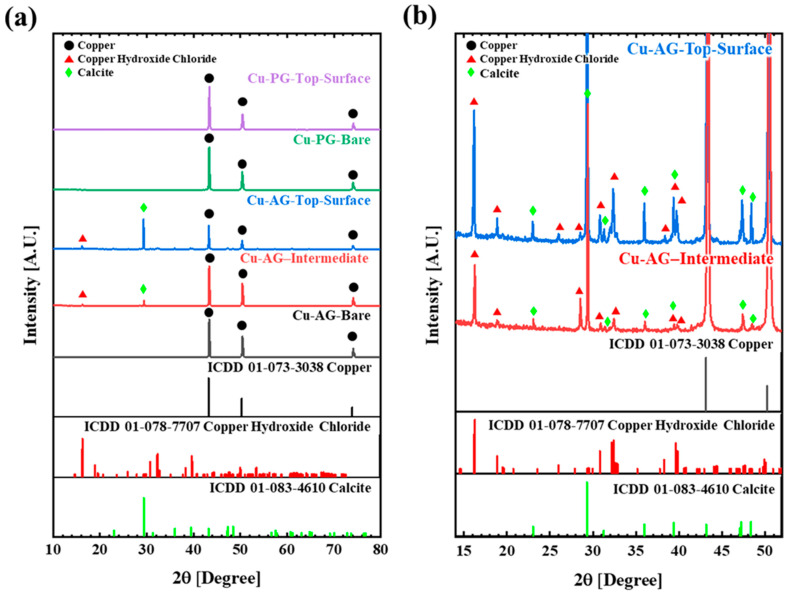
(**a**) X-ray diffraction patterns (XRD) of the OFC samples after the immersion test in AG and PG at 70 °C for three weeks and (**b**) a magnified version of Cu-AG for the intermediate layer and top surface layer.

Following the immersion experiment, an analysis was conducted through SEM and EDS (Figure 3 and Figure 4) and XRD (Figure 5) to elucidate the morphology, composition of the corrosion products, and state of the formed layer.

In Figure 3, the surface images display the corrosion product layer with and without etching in the AG environment. Figure 4 presents the equivalent results conducted in a PG environment. Figure 3a displays the SEM image of corrosion products formed in the outermost surface layer, featuring unevenly stacked, large, crystallized products. In the image of the material where excessive corrosion products were removed through etching (Figure 3c), the corrosion of the Cu material progressed, accompanied by the presence of densely sized particles and micron-scale cracks. Elemental mapping revealed the composition of the corrosion products formed in the substrate and surface layer. The chemical composition (atomic ratio) of the corroded OFC in AG or PG conditions is summarized in Table 3. The base layer prominently featured Cu and Mg, whereas the surface layer primarily comprised Cu, O, Cl, and Ca elements. Hence, Cu and Mg compounds (oxides, sulfides, and chlorides) were initially formed, influenced by the reactivity of Mg and Ca cations, which are more active than copper. As corrosion proceeded, additional Ca-based compounds appeared to emerge.

In contrast, the electron microscope image and elemental map presented in Figure 4 predominantly revealed Cu and O, with notably high oxygen content in the layer containing corrosion products and shallow oxygen content in the base layer. This suggested the formation of a uniform layer, which was anticipated to make a substantial contribution to corrosion stability.

For a comprehensive understanding of the corrosion product formation, an XRD pattern analysis was conducted for each point, as depicted in Figure 5. The OFC exposed to the PG environment exhibited characteristics corresponding to pure copper. In contrast, specimens exposed to the AG environment did not reveal Mg-related compounds, but instead, the presence of copper hydroxide chloride and calcite was confirmed [18]. Concerning the calcite material, it was observed within the middle layer (moderately etched), with the intensity of the representative peak increasing as it approached the outermost layer, signifying an augmented material quantity. This observation aligned with the trends established in the previous SEM–EDS analyses.

**Figure 6:**
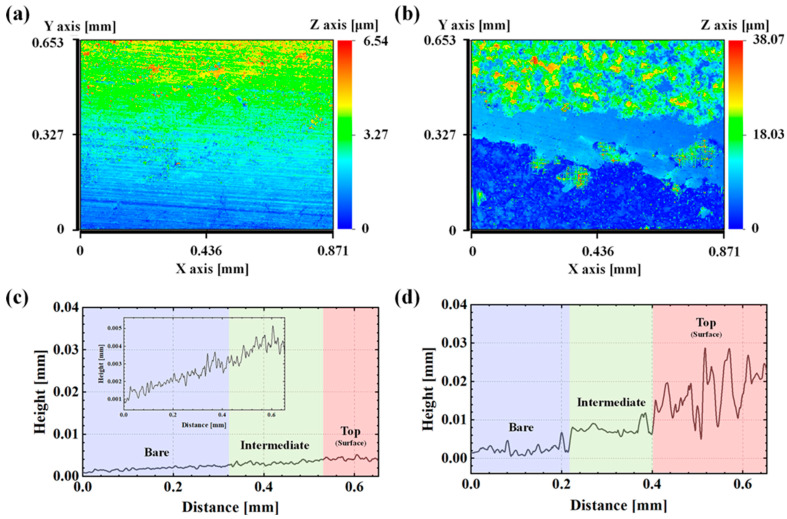
(**a**,**c**) Surface topography of OFC after the immersion test in PG for three weeks. (**b**,**d**) Surface topography of OFC after the immersion test in the AG for three weeks.

The surface topography analysis of the oxygen-free copper (OFC) sample following the immersion test was performed using a white interferometer, as illustrated in Figure 6. Only half of the specimen was etched to examine the thickness and roughness of the corrosion products formed, and then measurement was conducted. Table 4 provides a summary of the R_a_ values at the bare and top positions of the corroded OFC specimens.

As depicted in Figure 6a, very thin and uniform corrosion products were evident on the OFC exposed to the PG, extending from the base layer to the surface layer. The thickness of the corrosion product formed after the 3-week immersion test was measured to be approximately 240 nm, while in the case of the AG, the thickness amounted to 3.6 μm. The roughness profile of the top layer revealed significant peaks and valleys, serving as further evidence of the heterogeneity of the formed corrosion products.

Summarizing these analytical results, it can be observed that in the case of the AG, which had high ion concentrations, the formation of corrosion products like Cu_2_Cl_3_OH on the surface due to the combined chemical action of oxygen and chloride led to a significant corrosion rate. However, in the PG environment, a stable CuO was formed, inhibiting corrosion. This demonstrated that the corrosion behavior of OFC varied significantly depending on the ion concentration and the composition of the groundwater. Therefore, it highlighted the importance of groundwater environment as a crucial factor in the site selection for future deep geological repositories.

## 4. Conclusions

In this study, after a thorough examination encompassing a series of immersion tests, electrochemical analyses, SEM and EDS characterizations, XRD pattern analyses, and white interferometer evaluations, the corrosion behavior of OFC under diverse groundwater environments was investigated.

The results revealed that the corrosion behavior of OFC was profoundly influenced by factors such as the type of groundwater and the temperature of the environment. In the high-temperature AG environment, OFC displayed heightened corrosion susceptibility, leading to a notably accelerated corrosion rate. This was corroborated by the observed changes in surface color (from light brown to black to blue) and the formation of Cu_2_Cl_3_OH and calcite (CaCO_3_) in the AG environment.

Conversely, exposure to the PG environment led to more stable corrosion behavior, with corrosion products being predominantly comprised of uniform CuO. However, the rise in temperature led to a 3.7-fold escalation in the corrosion rate, even in the Korean-collected groundwater. The pronounced disparity in corrosion tendencies between the artificial groundwater (AG) and procured groundwater (PG) was attributed to the considerably lower cation/anion concentration, ranging from 1/15 to 1/10. This pronounced dissimilarity in corrosion patterns between the AG and PG could be ascribed to the substantial difference in the cation-to-anion concentration ratios, which ranged from 1/15 to 1/10.

Additionally, the surface topography analyses using a white interferometer revealed a significant difference in the thickness and roughness of the corrosion products between the AG and PG environments. The PG environment exhibited a remarkably thinner and more uniform corrosion product layer, underscoring its enhanced corrosion stability.

These findings underscored the intricate interplay between environmental factors, such as groundwater type and temperature, and their impact on the corrosion behavior of OFC. The comprehensive understanding gained from this study provides valuable insights into the performance and stability of copper materials in diverse disposal environments. Future research will delve deeper into elucidating key factors in this corrosion process, enabling us to further refine our understanding and contribute to developing more robust corrosion-resistant materials for long-term disposal applications.

## Figures and Tables

**Table 1:** Characteristics of groundwater at the Olkiluoto site, Olkiluoto-simulated artificial groundwater, and procured groundwater (South Korea).

	Olkiluoto Groundwater * [10]	Artificial Groundwater (AG)	Procured Groundwater *^,^ ** (PG)
pH	7.6–8.1	7.81	7.02
Na^+^	4800	4452	31.1
K^+^	20.00	19.9	3.17
Ca^2+^	4800	4809	42.4
Mg^2+^	70.00	70.48	9.36
NH^4+^	0.05	0.05	-
Mn^2+^	-	-	0.429
Cl^−^	16,000	15,598	21.6
HS^−^	0.50	0.18	-
NO_2_^−^	0.02	-	<0.1
SO_4_^2−^	40.00	38.40	106
Br^−^	-	-	<0.1
SiO_2_	-	-	55.7
CH_4_ (g)	130–400	-	-
H_2_ (g)	<0.4	-	-

* The depth of the Olkiluoto groundwater and procured groundwater (PG) was 400–600 m and 210 m, respectively. ** The PG was collected within the underground research facility constructed for the safety assessment of radioactive waste disposal in South Korea.

**Table 2:** Potentiodynamic polarization (PDP) parameters for the OFC corrosion in different groundwaters and temperatures.

	Potential (V_Hg/HgO_)	Current Density (μA/cm^2^)	β_a_	β_c_
Cu-AG-L	−0.49	2.90	0.33	0.21
Cu-AG-H	−0.17	30.90	29.84	0.29
Cu-PG-L	−0.16	3.56	0.18	0.68
Cu-PG-H	−0.08	13.30	2.26	1.92

**Table 3:** The chemical composition (At. %) of the corroded OFC in AG or PG conditions with EDS.

	Cu	O	Ca	Cl	Mg	S	Total
Cu-AG-H (outermost)	19.89	55.49	12.74	10.43	0.91	0.54	100
Cu-AG-H (etched)	28.57	61.47	0.08	0.03	8.83	0.72	100
Cu-PG-H (outermost)	49.67	50.33	-	-	-	-	100
Cu-PG-H (etched)	97.55	2.45	-	-	-	-	100

**Table 4:** Average surface roughness of the corroded OFC in AG or PG conditions.

	Cu-PG-H (Top)	Cu-PG-H (Bare)	Cu-AG-H (Top)	Cu-AG-H (Bare)
Ra (μm)	0.477	0.235	5.120	1.550

## Data Availability

The data presented in this study are available on request from the corresponding author.

## Supplementary Materials

The following supporting information can be downloaded at: https://www.mdpi.com/article/10.3390/ma17010074/s1, Figure S1: (a) the OCP plot, (b,c) the equivalent circuit utilized for the EIS fitting; Figure S2: Visual representations of the groundwater solution before and after the immersion test (a) AG solution and (b) PG solution.
