# Supplementary material for: Investigation of Corrosion Behavior of Oxygen-Free Copper Canisters in Groundwater Chemistry of Deep Geological Repositories
Source: Materials (Basel). 2023 Dec 22;17(1):74. doi: 10.3390/ma17010074 (PMC10779650; doi:10.3390/ma17010074)
Supplement: Supplementary file 1 [file materials-17-00074-s001.zip › materials-2725226-supplementary.pdf]

Supplementary

1. Open Circuit Potential (OCP) plot for Artificial Groundwater (AG) and Procured Groundwater (PG), along with the equivalent circuit for Electrochemical Impedance Spectroscopy (EIS) fitting.

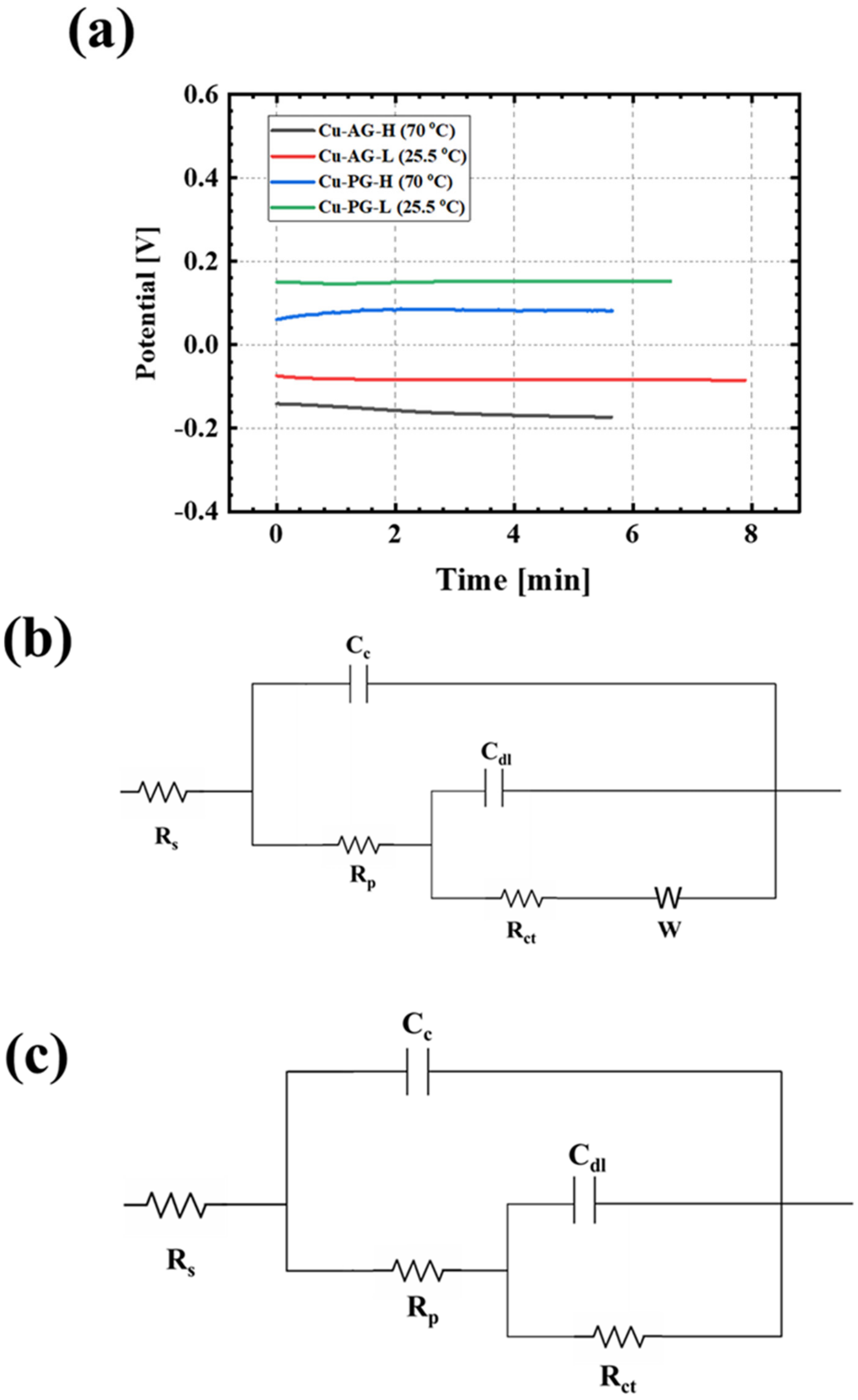

Figure S1. (a) the OCP plot, (b and c) the equivalent circuit utilized for the EIS fitting.

2. *Visual representations of the groundwater solution before and after the immersion test*

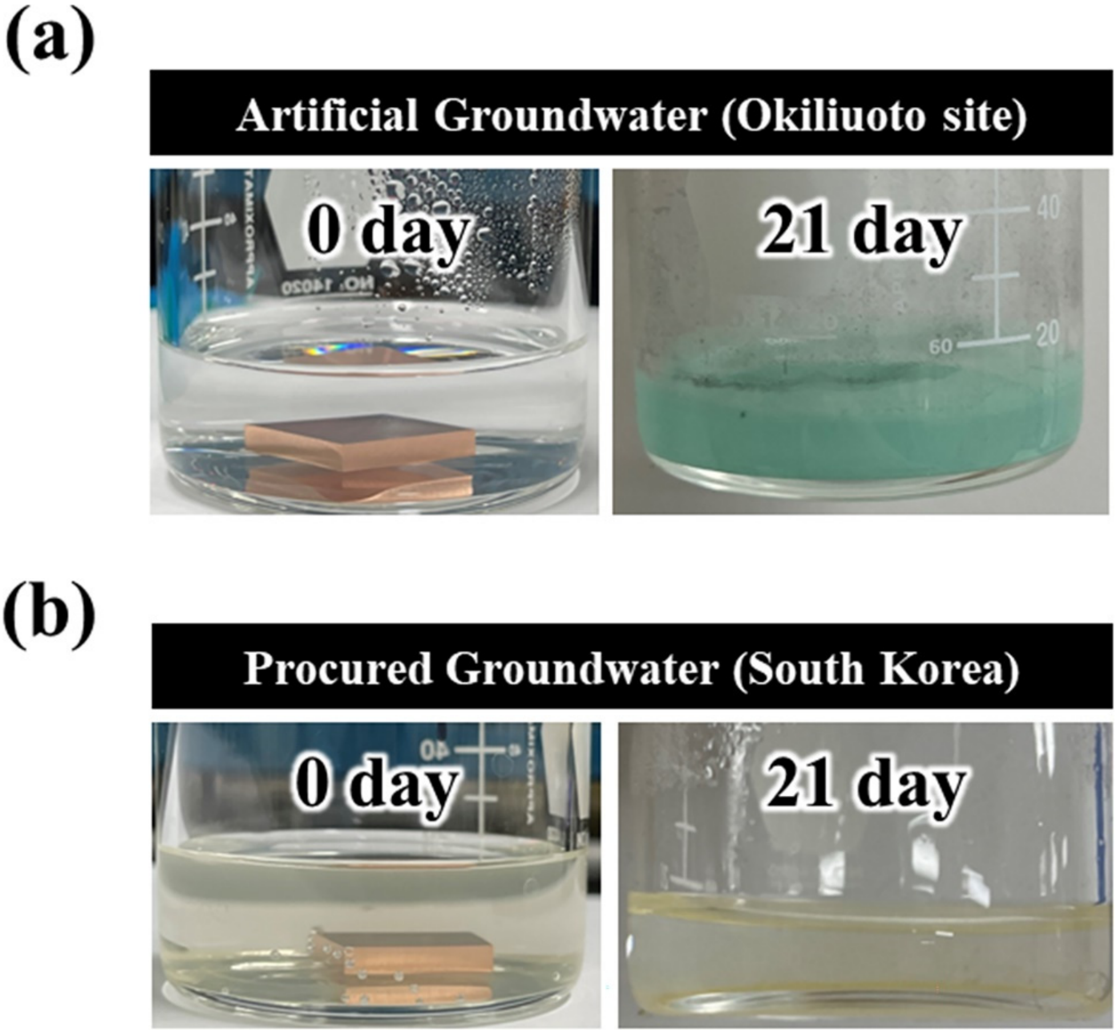

Figure S2. Visual representations of the groundwater solution before and after the immersion test (a) AG solution and (b) PG solution.
